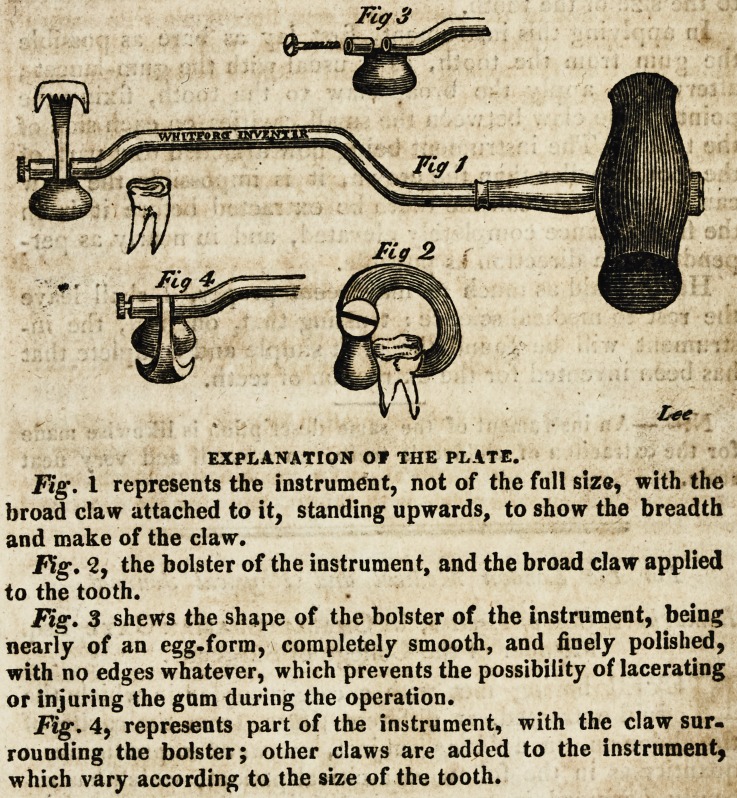# Description of a New Instrument for Extracting Teeth

**Published:** 1816-06

**Authors:** J. Whitford

**Affiliations:** Surgical Instrument Maker, 47, West Smithfield.


THE LONDON
### Medical and Physical Journal.

6 OF VOL. XXXV.] JUNE, 1816. [NO. 208.

"For many fortunate discoveries in medicine, and for the detection of numerous errors, the world is indebted to the rapid circulation of Monthly Journals; and there never existed any work to which the Faculty in EUROPE and AMERICA were under deeper obligations than to the Medical and Physical Journal of London, now forming a long, but an invaluable, series."—RUSH.

*For the London Medical and Physical Journal.*

### *Description of a New Instrument for extracting Teeth;* by Mr. J. WHITFORD, Surgical Instrument Maker, 47, West Smithfield.

AFTER the many attempts at improving the instrument now in use for the extraction of teeth, it is to be feared that any fresh proposal will be received with much difficulty. Still,

Still, however, I have many reasons to hope, that the one now submitted to the faculty will be found to work with less pain to the patient and greater facility to the operator than any hitherto invented. The idea is simple, being derived from the manner in which a nail driven into a piece of wood is drawn out by pincers, or by the carpenter's claw-hammer. The claw embraces not the top of the nail but the body, and when fixed, the other end of the hammer serves as a fulcrum. By these means the nail is readily drawn out. Such is the case with the instrument now offered. The shank is as in the customary instruments; but the fulcrum, or bolster, is made of an egg-form, extremely smooth, to prevent the possibility of the gums being injured by any cutting edges as sometimes happens. It is well known that there is a cavity between every tooth in the jaw;—the claws of the instrument are therefore made of different breadths, to adapt themselves to the size of the tooth.

In applying this instrument, first lay as bare as possible the gum from the tooth, as is usual with the gum-lancet; afterwards, apply the broad-claw to the tooth, fixing the point of the claw between the small cavities on each side of the tooth. The instrument being now attached to a part of the tooth smaller than the crown, it is impossible the claw can slip off; nor can the tooth be extracted before it is in the first instance completely elevated, and in nearly as perpendicular a direction as possible.

Having said as much as may seem proper, I shall leave the rest to medical science; trusting that, on trial, the instrument will be found the most simple and complete that has been invented for the extraction of teeth.

---

**N.B.—An instrument of the same description is likewise made for the extraction of children's teeth, on a small and very neat scale.**

---

*For the London Medical and Physical Journal.*

## Figures and Tables

**Fig 3 Fig 1 Fig 4 Fig 2 f1:**